# HIV serostatus knowledge and serostatus disclosure with the most recent anal intercourse partner in a European MSM sample recruited in 13 cities: results from the Sialon-II study

**DOI:** 10.1186/s12879-017-2814-x

**Published:** 2017-11-25

**Authors:** Ulrich Marcus, Susanne Barbara Schink, Nigel Sherriff, Anna-Marie Jones, Lorenzo Gios, Cinta Folch, Torsten Berglund, Christiana Nöstlinger, Marta Niedźwiedzka-Stadnik, Sonia F. Dias, Ana F. Gama, Emilia Naseva, Ivailo Alexiev, Danica Staneková, Igor Toskin, Daniela Pitigoi, Alexandru Rafila, Irena Klavs, Massimo Mirandola, the Sialon II Network, Massimo Mirandola, Lorenzo Gios, Stefano Benvenuti, Ruth Joanna Davis, Massimo Lunardi, Silvana Menichelli, Michele Breveglieri, Martina Furegato, Wim Vanden Berghe, Peter de Groot, Christiana Nöstlinger, Veronica van Wijk, Katrien Fransen, Tine Vermoesen, Michiel Vanackere, Fourat Benchikha, Sandra Van den Eynde, Boris Cruyssaert, Mark Sergeant, Karel Blondeel, Pieter Damen, François Massoz, Erwin Carlier, Michael François, Stephen Karon, Safia Soltani, Thierry Martin, Alan De Bruyne, Françoise Bocken, Myriam Dieleman, Ivailo Alexiev, Reneta Dimitrova, Anna Gancheva, Dobromira Bogeva, Maria Nikolova, Mariya Muhtarova, Todor Kantarjiev, Viara Georgieva, Emilia Naseva, Petar Tsintsarski, Hristo Taskov, Tonka Varleva, Elena Birindjieva, Aneliya Angelova, Manol Antonov, Ulrich Marcus, Susanne Barbara Schink, Sandra Dudareva-Vizule, Matthias an der Heiden, Sami Marzougui, Viviane Bremer, Andrea Kühne, Kerstin Schönerstedt-Zastrau, Ruth Zimmermann, Andreas Wille, Kai Eckstein, Norman Buch, Philipp Moskophidis, Marc Grenz, Danilo Schmogro, Giuseppe Cornaglia, Antonella Zorzi, Elisabetta Tonolli, Giuliana Lo Cascio, Teresa Todeschini, Manuela Recchia, Lorella Pattini, Maria Rocca, Alessandra Bighignoli, Anita Galardi, Loredana Martini, Sandro Caffi, Pier Paolo Benetollo, Francesco Cobello, Chiara Bovom, Umberta Benvenuti, Giulia Bisoffi, Oscar Bortolami, Laura Crestani, Fabiano Comperini, Ercole Concia, Emanuela Lattuada, Massimiliano Lanzafame, Stefania Leonardi, Paola Del Bravo, Maddalena Cordioli, Fabio Rigo, Emanuele Guardalben, Ivan Marchesoni, Barbara Suligoi, Vincenza Regine, Lucia Pugliese, Saulius Caplinskas, Irma Caplinskiene, Rima Krupenkaite, Gediminas Sargelis, Arturas Rudomanskis, Sónia Dias, Ana Gama, Oriana Brás, João Piedade, Ricardo Fuertes, Nuno Pinto, João Brito, Júlio Esteves, Jesus Rojas, Fernando Ferreira, Miguel Rocha, Hugo Machado, Maria José Campos, Luís Mendão, Magdalena Rosińska, Bożena Kucharczyk, Marta Niedźwiedzka-Stadnik, Łukasz Henszel, Andrzej Zieliński, Michał Czerwiński, Michał Pawlęga, Ewelina Burdon, Małgorzata Gajdemska, Agnieszka Guściora, Nikodem Klasik, Katarzyna Rżanek, Michał Sawicki, Michał Tęcza, Mateusz Dębski, Anna Maciejewska, Izabela Pazdan, Alexandru Rafila, Daniela Pitigoi, Adrian Abagiu, Carolina Marin, Ioana Panzariu, Alexandru Miroiu, Madalina Popa, Monica Likker, Maria Georgescu, Galina Musat, Dan Cojocaru, Mihai Lixandru, Raluca Teodorescu, Danica Staneková, Monika Hábeková, Tatiana Drobková, Zuzana Chabadová, Soňa Wimmerova, Maria Mojzesová, Filip Kunč, Michal Skurák, Peter Bodnar, Katarína Horniaková, Mária Krahulcová, Jarmila Präsensová, Martin Smoleň, Peter Záhradník, Pavol Tibaj, Irena Klavs, Tanja Kustec, Claudia Adamič, Mario Poljak, Robert Krošelj, Jana Mlakar, Miran Šolinc, Cinta Folch, Laia Ferrer, Alexandra Montoliu, Jordi Casabona, Anna Esteve, Montserrat Galdon, Victoria Gonzalez, Rafael Muñoz, Maria Axelsson, Torsten Berglund, Sharon Kuhlmann-Berenzon, Achilleas Tsoumanis, Inga Velicko, Christer Janson, Bartek Lindh, Kajsa Aperia, Buddha Babulanam, Hans Carlberg, Malte Davidsson, Nedo Entenza Gutierrez, Viktor Hildingsson, Henrik Klasson, Moises Peña Ramos, Cristian Quintero Rojas, Sven-Olof Sandberg, Andreas Samuelson, Eric Sjöberg, Tommy Sjölund, Simon Svensson, Iván Valencia, Filip Garcia, Olov Lindblad, Jon Voss, Ronnie Ask, Anders Blaxhult, Maarit Maliniemi, Monica Ideström, Nils Blom, Nigel Sherriff, Christina Panton, Glynis Flood, Katrien Fransen, Tine Vermoesen, Ross Boseley, Marc Tweed, Jonathon Roberts, Cinthia Menel Lemos, Paolo Guglielmetti, Wolfgang Philipp, Matthias Schuppe, Andrew Amato, Irina Dinca, Karin Haar, Anastasia Pharris, Teymur Noori, Igor Toskin, Armando Seuc, Natalie Maurer, Lev Zohrabyan, Alexandrina Iovita, Maddalena Campioni, Patrick Noack, Rosanna Peeling, Lisa Johnston

**Affiliations:** 1 Department of Infectious Diseases Epidemiology, Robert Koch-Institute, Berlin, Germany; 2 University of Brighton, Health Sciences, Brighton, UK; 3 Mill View Hospital, Sussex Education Centre, Research & Development, Brighton, UK; 4 Department of Health, Verona University Hospital, CReMPE - Regional Coordination Centre for European Project Management, Verona, Veneto Region Italy; 5 Centre d’Estudis Epidemiològics sobre les Infeccions de Transmissió Sexual i Sida de Catalunya (CEEISCAT), Agència de Salut Pública de Catalunya (ASPC), Badalona, Spain; 6 CIBER Epidemiología y Salud Pública (CIBERESP), Barcelona, Spain; 7 Department of Monitoring & Evaluation, Public Health Agency of Sweden, Solna, Sweden; 8 Department of Public Health, Institute of Tropical Medicine, Antwerp, Belgium; 9 Department of Epidemiology, National Institute of Public Health, Warsaw, Poland; 10 Universidade Nova de Lisboa, Instituto de Higiene e Medicina Tropical, Global Health and Tropical Medicine, Lisbon, Portugal; 11 Ministry of Health, Program “Prevention and Control of HIV/AIDS”, Sofia, Bulgaria; 12 National Centre of Infectious and Parasitic Diseases, National Reference Laboratory of HIV, Sofia, Bulgaria; 13 Slovak Medical University, National Reference Centre for HIV/AIDS, Bratislava, Slovakia; 14 Department of Reproductive Health and Research, World Health Organization, Geneva, Switzerland; 15 University of Medicine and Pharmacy Carol Davila, Department Clinic 2, Epidemiology, Bucharest, Romania; 16 National Institute for Infectious Diseases “Prof Dr Matei Bals”, Bucharest, Romania; 17 Department of Microbiology, National Institute of Infectious Diseases “Prof Dr Matei Bals”, Bucharest, Romania; 18 National Institute of Public Health, Communicable Diseases Centre, Ljubljana, Slovenia; 19 Department of Health, Infectious Disease Section, Verona University Hospital, CReMPE - Regional Coordination Centre for European Project Management, Verona, Veneto Region Italy

**Keywords:** Men who have sex with men, Bio-behavioural survey, HIV serostatus disclosure, HIV exposure

## Abstract

**Background:**

Knowledge of HIV status can be important in reducing the risk of HIV exposure. In a European sample of men-who-have-sex-with-men (MSM), we aimed to identify factors associated with HIV serostatus disclosure to the most recent anal intercourse (AI) partner. We also aimed to describe the impact of HIV serostatus disclosure on HIV exposure risks.

**Methods:**

During 2013 and 2014, 4901 participants were recruited for the bio-behavioural Sialon-II study in 13 European cities. Behavioural data were collected with a self-administered paper questionnaire. Biological specimens were tested for HIV antibodies. Factors associated with HIV serostatus disclosure with the most recent AI partner were examined using bivariate and multilevel multivariate logistic regression analysis. We also describe the role of serostatus disclosure for HIV exposure of the most recent AI partner.

**Results:**

Thirty-five percent (*n* = 1450) of the study participants reported mutual serostatus disclosure with their most recent AI partner or disclosed having HIV to their partner. Most of these disclosures occurred between steady partners (74%, *n* = 1077). In addition to the type of partner and HIV diagnosis status, other factors positively associated with HIV serostatus disclosure in the multilevel multivariate logistic regression model were recent testing, no condom use, and outness regarding sexual orientation. Disclosure rates were lowest in three south-eastern European cities.

Following condom use (51%, *n* = 2099), HIV serostatus disclosure (20%, *n* = 807) was the second most common prevention approach with the most recent AI partner, usually resulting in serosorting. A potential HIV exposure risk for the partner was reported by 26% (111/432) of HIV antibody positive study participants. In 18% (20/111) of exposure episodes, an incorrect HIV serostatus was unknowingly communicated. Partner exposures were equally distributed between steady and non-steady partners.

**Conclusions:**

The probability of HIV exposure through condomless AI is substantially lower after serostatus disclosure compared to non-disclosure. Incorrect knowledge of one’s HIV status contributes to a large proportion of HIV exposures amongst European MSM. Maintaining or improving condom use for anal intercourse with non-steady partners, frequent testing to update HIV serostatus awareness, and increased serostatus disclosure particularly between steady partners are confirmed as key aspects for reducing HIV exposures amongst European MSM.

**Electronic supplementary material:**

The online version of this article (10.1186/s12879-017-2814-x) contains supplementary material, which is available to authorized users.

## Background

Human Immunodeficiency Virus (HIV)-testing is promoted amongst people at increased risk of HIV infection mainly to enable early diagnosis and treatment for those found HIV positive, but also to support HIV/AIDS literacy and ultimately behaviour change [1]. The impact of HIV test results on sexual behaviour and transmission risk behaviour has more frequently been analysed for people diagnosed with an HIV infection than for people receiving a negative HIV test result [2, 3]. Findings regarding the association between serostatus disclosure and sexual risks amongst MSM have been inconsistent [4]: disclosure of a positive HIV serostatus is often associated with a reduced risk for HIV transmission either because no anal intercourse (AI) takes place, or through subsequent condom use if serodiscordant, or through condomless anal intercourse (CLAI) if seroconcordant (condom serosorting). The risk for other sexually transmitted infections (STIs) however, may increase if HIV serostatus disclosure leads to CLAI [5]. The consequences of seronegative status disclosure on HIV transmission risk remain less clear [6–8].

A substantial body of literature on the enabling and hindering factors for HIV status disclosure between MSM has been published, identifying the following factors associated with disclosure of HIV serostatus: disclosure is more likely between steady partners compared to non-steady partners; and for non-steady partners, disclosure depends on the setting where the partners have met. HIV positive status disclosure is negatively associated with perceived HIV-related stigma, depressive symptoms, and increasing age (if older than 50 years) [4, 9–18].

A negative HIV test result is often used to guide decisions on condom use for AI,particularly in the context of mutual HIV serostatus disclosure with steady and, to a lesser degree, with non-steady partners [19]. Disclosure of the last HIV test result can be inaccurate if HIV seroconversion occurred after the test result was received, which may then result in unintended HIV exposure of assumed seroconcordant HIV negative partners. In this present study, we term this “failed serosorting”. The frequency with which failed serosorting occurs depends on the frequency and type of partners for CLAI after having received a negative HIV antibody test result, testing frequency, and the reliability of HIV serostatus information communicated by sexual partners. Serostatus disclosure can be compromised by people disclosing a negative HIV status despite having never been tested; a phenomenon repeatedly observed but rarely analysed and reported in MSM surveys [19, 20].

A major advantage of bio-behavioural surveys compared to behavioural surveys is the possibility to analyse the accuracy of assumed HIV serostatus. Furthermore, they also allow the effects of condom use decisions following serostatus disclosure on HIV exposure risks to be explored, particularly when analysing serostatus disclosure to participants’ last AI partner(s).

There are two objectives we aim to address with the present paper. First, we aim to describe the frequency of HIV serostatus disclosure and to identify factors associated with HIV serostatus disclosure to the most recent AI partner in a multi-city European MSM sample. Second, based on self-reported behaviours, we aim to examine the effects of serostatus disclosure in relation to other risk management tactics on HIV exposure risks for sexual partners of study participants with HIV antibodies during the most recent AI episode in the study population.

## Methods

### Study procedures

Study participants were recruited to the European bio-behavioural Sialon-II study using Time-Location Sampling (TLS) in nine cities (Barcelona, Brighton, Brussels, Hamburg, Lisbon, Ljubljana, Sofia, Stockholm, and Warsaw) and Respondent-Driven Sampling (RDS) in four cities (Bratislava, Bucharest, Verona, and Vilnius).Participants were eligible for enrolment if they were: males aged 18 years or older, and; had had sex with at least one male partner in the previous 12 months, and; gave their consent to all study procedures including filling out an anonymous self-administered questionnaire and providing a biological sample for testing. Recruitment methods, study procedures, questions asked, and sample collection and testing have been published elsewhere [21]. The study protocol was approved by the World Health Organization (WHO) Research Project Review Panel (RP2) and the WHO Research Ethics Review Committee (WHO-ERC), and by ethics review committees in all participating countries.

In TLS cities, participants were recruited during 2013, whilst in RDS cities recruitment started in 2013 and finished in 2014.

### Measures

In this article, we focus on participant characteristics, societal attitudes experienced by gay and bisexual men, as well as relationships and behaviours with the most recent AI partner. The timing of the most recent AI event was not elicited. To measure the outcome ‘serostatus disclosure to the most recent AI partners’ a variable was constructed based on respective questions on HIV serostatus communication with the last AI partner. There were two questions: 1) What did you tell your last AI partner about your HIV serostatus? and 2) What did you think about the HIV serostatus of your last AI partner before you had sex? The response options for 1) being: I told him I don’t know; I am HIV-negative; I am HIV-positive; I said nothing; I don’t remember; I refuse to answer. The response options for 2) being: I assumed he is HIV-negative/positive; I knew he is HIV-negative/positive; I knew he didn’t know his status; I didn’t think about; I don’t remember; I refuse to answer. The constructed variable distinguishes between successful serostatus disclosure (i.e. a communication that establishes HIV serostatus concordance or discordance, including unilateral HIV infection disclosure), and unsuccessful serostatus disclosure (i.e. a communication where either none or only one of the involved partners disclosed his serostatus, with the exception of unilateral HIV infection disclosure, see above). Respondents indicated whether the most recent AI partner was a steady or non-steady partner (steady included ‘boyfriends’ and ‘husbands’, and excluded ‘sex buddies’; see Additional file 1 ‘Study questionnaire’).

Mutual disclosure was assumed if the respondent had both disclosed his status to his partner and indicated *knowing* the status of his partner prior to engaging in AI. If AI was reported with a steady partner, we cannot assume that the most recent AI was the first occasion to communicate about HIV status; therefore, the definition for serostatus disclosure with steady partners was broadened to include those who indicated knowledge of their partners’ HIV serostatus and reported a concordant last HIV test result, but did not explicitly confirm that they had disclosed their serostatus to their steady partner at this most recent AI episode.

Those reporting more than one partner during the most recent AI were included, but criteria for categorisation of disclosures were stricter because the questionnaire was not designed to assess behaviours with multiple partners (see Additional file 2).

The second outcome is a purely quantitative description of risk management tactics used with the participant’s most recent AI partner, including the assessment whether the partners of those who are HIV antibody positive have potentially been exposed to HIV. Here, the self-reported risk management tactics which are serostatus-dependent are contrasted with the HIV antibody test result of a self-collected oral fluid specimen (collected in TLS cities) or a blood specimen (collected in RDS cities). The test result could confirm or contradict the assumed HIV status, i.e. self-reported serostatus was qualified as either accurate or inaccurate. A hierarchy of six risk management tactics (including those not requiring communication, those requiring communication, and a lack of discernible risk management) were defined with the following order: 1) condom use; 2) treatment with anti-retroviral therapy (ART) to maintain an undetectable viral load; 3) true serostatus concordance (assuming that reported serostatus of the partner was correct); 4) true serostatus discordance (i.e. both partners aware of HIV discordance and not reporting condom use or undetectable viral load); 5) falsely assumed serostatus concordance (i.e. self-report of a negative HIV test result and disclosing this result to their most recent AI partner although HIV antibodies were detected in the specimen provided for testing on the day of the study participation; alternatively, this could also be due to having been infected by this most recent AI partner, in which case this partner and not the respondent falsely assumed serostatus concordance; 6) none of the risk reduction tactics described above whilst reporting CLAI with HIV antibodies detected in the specimen (e.g. a participant who reported CLAI with his most recent non-steady AI partner but without communicating his HIV status). If a participant was HIV-positive, the last three risk management tactics were assumed to have potentially exposed their most recent AI partner to HIV, and were stratified by type of partner into men disclosing correctly their actual HIV status, men disclosing (unknowingly) an incorrect HIV status, men who did not communicate with their partner about their HIV status or who did not provide this information, and men for whom we were unable to determine their HIV status awareness.

### Statistical analysis

For the first outcome variable, factors associated with successful HIV serostatus disclosure with the last AI partner were identified using a two-level multilevel logistic regression model (MLM) with individuals nested within city and a random intercept for city. The random component accounts for locational clustering. To explore potential explanatory variables for entry into the models, bivariate analyses were used to assess the associations between individual variables and successful HIV serostatus disclosure. Variables included in the MLM model were those with a statistically significant association (*p*-value <0.05) in the bivariate analysis. Model selection for the MLM used the forward approach and variables were retained if p-value <0.10. As each variable was added, the new model was compared to the nested model using a likelihood ratio test. Significance was determined by *p* < 0.05.

The final MLM estimated the odds ratio (OR) and the corresponding 95% confidence interval (95% CI) for factors statistically significantly associated with successful serostatus disclosure. Stata® Version 14 was used for all analyses.

The following explanatory variables were analysed: age (categorised in 10-year age groups), migration status (whether living in a country other than the country of birth), education (categorised as secondary or lower, high school diploma to post-secondary, university or higher), frequency of visiting gay venues with the possibility of sex-on-premises in the last 3 months (no, low or high), outness (the degree to which people are open about their sexual attraction with family members, friends and co-workers, categorised as open to nobody or few, to less than half, to more than half, to all or almost all), type of partner during the most recent AI episode (steady, non-steady), condom use during the most recent AI episode (condom use, no condom use), HIV status awareness, (self-reported) viral load if HIV positive (detectable, undetectable), recency of the last HIV test (last 12 months, >12 months ago, or never tested), sexual role during the most recent AI episode (receptive, insertive, both), and substance use in the context of the most recent AI episode (no substance, one-to-two substances, more than two substances). We also hypothesised the following: 1) that a recent negative HIV antibody test result, as well as a recent viral load test below the detection limit, would both increase the intention to disclose these results to a sexual partner and; 2) not having been tested for HIV antibodies and being diagnosed with HIV and having a detectable viral load would decrease the likelihood of disclosing this to potential sexual partners. Thus we constructed a variable distinguishing between men reporting a recent (<12 months) negative HIV antibody test or a positive HIV antibody test with a recent viral load below the limit of detection, and the other reported possibilities, such as no history of previous HIV testing or a positive HIV antibody test without reporting a viral load below the limit of detection. Perceived stigma towards gay/bisexual people was assessed using a 5-point Likert scale ranging from 1 (very negative) to 5 (very positive) regarding respondents’ perceptions of homophobia across three domains: 1) work/school; 2) parents, and; 3) friends/acquaintances (Cronbach’s alpha = 0.73). The points from the three dimensions were added for a score: 1–7.5 points were defined as “negative attitudes”, 8–10.5 points as “neutral attitudes”, and 11–15 points as “positive attitudes”. To account for missing values the Predictive Mean Matching (PMM) was used. Three variables related to the perceived attitude towards gay and bisexual individuals in social contexts were imputed: the perceived attitude towards gay and bisexual men at school/work (missing *n* = 182/4091), the perceived attitude from parents or family members (missing *n* = 301/4091), and the perceived attitude from friends or acquaintances (missing *n* = 236/4091). The procedure used as predictors city, education and outness. PMM as a semi-parametric imputation approach allows imputed values to be more plausible when the normality assumption is violated.

To summarise HIV risk management tactics and HIV exposure risk, descriptive statistics (counts and proportions) where used. HIV exposure risk was analysed exclusively for the last AI partners of study participants with a biological specimen in which HIV antibodies had been detected. Participants who themselves did not have HIV and reported a last AI partner with HIV were not considered because information on treatment and viral load communication with the last AI partner was not collected.

No sampling weights were applied during statistical analysis because we wanted to model the relationship between explanatory variables and the outcome ‘serostatus disclosure to the last AI partner’ and did not intend to generalise the result to the specific study populations in the different cities.

## Results

### Study sample

We recruited 4901 participants in the 13 study cities, with 3596 participants from the 9 TLS cities, and 1305 from the 4 RDS cities. Median age was 32 years (IQR: 26–41 years), 12% (*n* = 456) of the participants were immigrants (born abroad and living in the study country), 5% (*n* = 213) were visitors (born abroad and visiting in the study country). HIV antibodies were detected in the biological specimens of 497 study participants. A previous HIV diagnosis was reported by 234, a previous negative HIV test result or no previous HIV test were reported by 163, and for 100 study participants it was not possible to determine their HIV status awareness due to missing responses. A detailed description of the study sample has been published in the study report [22].

### Missing values

The proportion of missing values ranged from 0.1% (*n* = 5) for age to 26% (*n* = 1292) for condom use with the most recent AI partner. Data were missing on measured HIV antibody status or self-reported serostatus knowledge for 7% (*n* = 320) of respondents. For most of the items analysed in this paper, responses were available from approximately 4000 participants (range: 3609–4110; see Table 1). The final MLM which included the condom use variable was based on 3219 observations.

**Table 1:** Association of demographic, behavioural, psychosocial and biological factors with HIV serostatus disclosure to the most recent anal intercourse partner amongst MSM in 13 European cities, Sialon-II bio-behavioural survey, 2013–2014

		Proportion with HIV serostatus disclosure at the last AI	Odds Ratio	95% Confidence Interval		p-value^
Age group [years]	18–24	31.6%	**0.82**	0.68	0.97	0.024
	25–34	36.1%	ref.			
	35–44	39.5%	1.15	0.98	1.36	0.092
	45–54	32.9%	0.87	0.70	1.07	0.195
	55+	31.3%	0.80	0.59	1.10	0.169
	*Total*	*35.3%*				*0.0040*
Education	secondary school or lower	28.1%	**0.66**	0.49	0.90	0.01
	high school diploma or post-secondary	33.1%	**0.84**	0.73	0.96	0.01
	university studies or higher	37.1%	ref.			
	*Total*	*35.1%*				*0.0035*
Migration status	native	34.1%	ref.			
	migrant	41.0%	**1.34**	1.13	1.59	<0.001
	*Total*	*35.2%*				
City	Hamburg	35.9%	ref.			
	Barcelona	37.0%	1.05	0.77	1.43	
	Bratislava (RDS)	26.4%	0.64	0.46	0.89	
	Brighton	46.9%	1.57	1.15	2.15	
	Brussels	43.4%	1.37	1.00	1.87	
	Bucharest (RDS)	22.8%	0.53	0.34	0.82	
	Lisbon	37.7%	1.08	0.80	1.47	
	Ljubljana	42.1%	1.30	0.95	1.77	
	Sofia	29.6%	0.75	0.55	1.02	
	Stockholm	39.0%	1.14	0.81	1.60	
	Verona (RDS)	29.6%	0.75	0.55	1.03	
	Vilnius (RDS)	33.4%	0.90	0.65	1.24	
	Warsaw	32.2%	0.85	0.62	1.16	
	*Total*	*35.3%*				
Frequency of gay sex venue attendance in recent 3 months	no	34.7%	ref.			
	low (1–3 times)	36.1%	1.06	0.90	1.24	0.49
	high (>3 times)	34.6%	0.99	0.84	1.18	0.95
	*Total*	*35.3%*				0.67
Perceived gay stigma	experienced positive attitudes (score points 11–15)	40.7%	ref.			
	experienced neutral attitudes (score points 8–10.5)	32.7%	**0.70**	0.61	0.81	<0.001
	experienced negative attitudes (score points 1–7.5)	26.4%	**0.53**	0.43	0.64	<0.001
	*Total*	*35.3%*				<0.001
Outness towards family, friends and co-workers	nobody/few	27.1%	ref.			
	less than half	31.8%	**1.25**	1.01	1.56	0.04
	more than half	35.8%	**1.50**	1.23	1.83	<0.001
	all/almost all	41.8%	**1.93**	1.64	2.28	<0.001
	*Total*	*35.3%*				<0.001
Number of CLAI partners in most recent 6 months	no partner	28.9%	ref.			
	1 partner	48.4%	**2.31**	1.96	2.72	<0.001
	2–5 partners	33.6%	**1.25**	1.05	1.48	0.01
	6–10 partners	26.5%	0.89	0.64	1.24	0.49
	>10 partners	32.4%	1.18	0.82	1.70	0.37
	*Total*	*35.6%*				<0.001
Type of partner during most recent AI	steady	54.1%	ref.			
	non-steady	17.2%	**0.18**	0.15	0.20	<0.001
	more than one	21.0%	**0.23**	0.16	0.31	<0.001
	*Total*	*35.3%*				<0.001
Condom use during most recent AI	condom use during last AI	26.6%	ref.			
	no condom use	47.8%	**2.52**	2.19	2.91	<0.001
	*Total*	*35.6%*				<0.001
HIV test during last 12 months	HIV test last 12 months	40.8%	ref.			
	never tested or tested >12 months ago	26.9%	**0.53**	0.47	0.61	<0.001
	*Total*	*34.9%*				<0.001
Negative HIV test result during last 12 months	no negative test result^b^	27.4%	ref.			
	negative antibody test	40.6%	**1.81**	1.58	2.06	<0.001
	negative viral load test	55.3%	**3.28**	2.36	4.56	<0.001
	*Total*	*35.3%*				<0.001
Most recent viral load measurement^a^	detectable viral load	60.6%	ref.			
	undetectable viral load	55.7%	0.82	0.38	1.75	0.60
	respondent did not know	28.3%	**0.26**	0.12	0.54	<0.001
	*Total*	*39.8%*				<0.001
Sexual role during most recent AI	insertive	35.3%	ref.			
	receptive	32.8%	0.89	0.76	1.05	0.17
	both	39.1%	1.17	1.00	1.38	0.05
	*Total*	*35.5%*				0.005
Number of substances used during most recent AI	no substances	39.7%	ref.			
	1–2 substances	31.8%	**0.71**	0.62	0.81	<0.001
	> 2 substances	32.1%	**0.72**	0.56	0.91	0.01
	*Total*	*35.3%*				<0.001
HIV status awareness	newly diagnosed	22.5%	ref.			
	negative	35.0%	**1.86**	1.24	2.79	<0.001
	already known	56.4%	**4.47**	2.76	7.23	<0.001
	*Total*	*35.8%*				<0.001

*RDS* Respondent Driven Sampling, *AI* anal intercourse, *CLAI* condomless anal intercourse, *OR* Odds Ratio. OR <1.0 indicates lower odds for disclosure, bold indicates ORs with a *p*-value <0.05

^The p-value from Wald test for categorical variables is reported on the variable’s Total row

^a^a previous HIV diagnosis was reported by 234 study participants; only these could report on the last viral load measurement

^b^ no negative test result includes men not tested for HIV antibodies, and HIV antibody positive men with detectable viral load

### Factors associated with HIV serostatus disclosure

Of those who answered the respective questions, successful serostatus disclosure to the most recent AI partner was reported by more than one third of study participants (35%, 1451/4110; see Table 1). HIV serostatus disclosure at the last AI was more common if the partner was a steady partner (54%) compared to a non-steady partner (17%); it was highest in the age group 35–44 years (40%) and higher amongst migrants (41%) than non-migrants (34%). Men with higher education were more likely to disclose their serostatus than those with secondary or lower levels. Compared with study participants who were out about their sexual orientation to ‘nobody or only a few people’, increasing proportions of those who were out to ‘less than half’, ‘more than half’, or ‘almost all or all’ of the people they knew disclosed their serostatus. Serostatus disclosure varied considerably between study cities with a North-West (high) to South-East (low) gradient. There were no differences regarding serostatus disclosure between respondents frequently visiting sex-on-premises venues in the last 3 months and gay venues without this option. It was more common amongst study participants reporting an HIV test in the last 12 months (41%) than amongst men who tested longer than 12 months ago or never (27%); it was also more common amongst men aware of having HIV (56%) than amongst men having received a negative HIV test result (35%). Serostatus disclosure was lowest amongst men reporting a last negative test result but testing positive for HIV antibodies in the specimen provided in the study (23%). Disclosure was most common if the respondents reported only one CLAI partner in the last 6 months, followed by the group with 2–5 CLAI partners. Compared to serostatus disclosure of men who were insertive (35%) during the most recent AI episode, a larger proportion of men reporting both receptive and insertive AI disclosed (39%), while serostatus disclosure was less common amongst men reporting only being receptive (33%). Likewise, serostatus disclosure was less common amongst men reporting condom-protected AI (27%) compared to CLAI (48%). The use of drugs in connection with the most recent AI was negatively associated with serostatus disclosure: participants reporting substance use during their last AI episode reported less serostatus disclosure than those who reported no substance use, but no dose-response effect (decreasing proportions who disclosed with increasing number of drugs consumed) was detected.

Table 2 shows the results of the final MLM to explore factors associated with successful HIV serostatus disclosure. Factors associated with increased successful serostatus disclosure were: having a recent negative HIV antibody test (OR = 2.69, 95% CI: 2.23, 3.23; *p* < 0.001), being out to all/almost all family, friends and co-workers (OR = 1.69, 95% CI: 1.32, 2.16; p < 0.001), already knowing HIV status (OR = 6.47, 95% CI: 3.37, 12.45; p < 0.001) and HIV risk management with the last AI partner other than condom use (OR = 1.80, 95% CI: 1.51, 2.14; p < 0.001). Factors associated with decreased success of serostatus disclosure were: having a non-steady partner (OR = 0.16, 95% CI: 0.14, 0.20; p < 0.001), having more than one partner (OR = 0.18, 95% CI: 0.12, 0.26; p < 0.001), having an as yet undiagnosed HIV infection (OR = 0.51, 95% CI: 0.31,0.86; *p* = 0.01) and if gay stigma was perceived as neutral (OR = 0.82, 95% CI: 0.68, 1.00; *p* = 0.04) or negative (OR = 0.72, 95% CI: 0.54, 0.97; *p* = 0.03). The likelihood ratio test comparing the final MLM with the equivalent single-level model indicated that they did not significantly differ (χ2(1) = 0.05, *p* = 0.41).

**Table 2:** Multilevel logistic regression: factors associated with HIV serostatus disclosure to the most recent anal intercourse partner amongst MSM in 13 European cities, Sialon-II bio-behavioural survey, 2013–2014

		Odds Ratio	95% Confidence Interval		p-Value
Type of partner during most recent AI					<0.001
	steady partner	ref.			
	non-steady partner	**0.16**	0.14	0.20	<0.001
	more than one partner	**0.18**	0.12	0.26	<0.001
Negative HIV test result during last 12 months					<0.001
	no HIV test result	ref.			
	negative antibody test	**2.69**	2.23	3.23	<0.001
	negative viral load test	0.63	0.30	1.33	0.23
Outness towards family members, friends, and co-workers					<0.001
	none or few	ref.			
	less than half	1.22	0.91	1.63	0.18
	more than half	1.32	1.00	1.73	0.05
	all/almost all	**1.69**	1.32	2.16	<0.001
HIV status awareness					<0.001
	not infected with HIV (tested or untested)	ref.			
	newly diagnosed	**0.51**	0.31	0.86	0.01
	already known	**6.47**	3.37	12.45	<0.001
Age group					0.0747
	18–24	0.80	0.64	1.00	0.051
	25–34	ref.			
	35–44	1.10	0.89	1.37	0.37
	45–54	0.84	0.63	1.14	0.26
	55+	0.79	0.51	1.24	0.31
Perceived gay stigma					0.04
	experienced positive attitudes (score points 11–15)	ref.			
	experienced neutral attitudes (score points 8–10.5)	0.82	0.68	1.00	0.04
	experienced negative attitudes (score points 1–7.5)	0.72	0.54	0.97	0.03
Condom use during most recent AI					<0.001
	condom use	ref.			
	no condom use	**1.80**	1.51	2.14	<0.001
_cons		0.46	0.34	0.62	<0.001
city		0.003	0,00	23.22	

Likelihood Ratio test vs. logistic regression: chibar^2^(1) = 0.05; Prob ≥ chibar^2^ = 0.4089

RDS = Respondent Driven Sampling; AI = anal intercourse; OR = Odds Ratio

OR <1.0 indicates lower odds for disclosure, bold indicates ORs with a p-value ≤0.05

### HIV risk management tactics in the study sample

We assessed the contribution of serostatus disclosure and erroneous serostatus disclosure to HIV exposure risks. For the definition of the risk management tactics we choose the perspective of the respondents, who may be unaware whether the serostatus they disclose is correct or not. They will usually know (1) whether condoms were used and (2) their HIV status if they have been diagnosed with HIV, and whether they take antiretroviral treatment.

Slightly more than half of the self-reported most recent AI episodes between study participants and their sex partners were protected by condom use and/or treatment (53% after exclusion of missing data; see Table 3); 27 % of participants reported CLAI without explicit serostatus disclosure, and 20% reported AI episodes where HIV-positive or HIV-negative seroconcordance had been established by mutual disclosure or HIV infection had been unilaterally disclosed.

**Table 3:** Hierarchy of HIV risk management by measured HIV status of the respondents and effects on HIV exposure of partners of HIV-positive respondents, Sialon-II bio-behavioural survey, 2013–2014

Hierarchy of risk management	Risk management	HIV-negative	Proportion excluding missing	HIV-positive	Proportion excluding missing	Total	Proportion excluding (including) missing	Partner exposure
1	condom use	*1878*		*146*		*2024*	50.9%	
1 + 2	condom use +treatment	n.a.		*75*		*75*		
2	treatment	n.a.		*92*		*92*		
Subtotal	disclosure-independent risk management	*1878*	*50.9%*	*313*	*72.5%*	*2191*	53.1% (44.7%)	
3 + 4	serostatus disclosure	*789*	*21.4%*	*18*	*8.8%*	*807*	19.6% (16.5%)	
3	serostatus concordance disclosure (correct)	HIV-negative serostatus concordance: *743*		HIV-positive serostatus concordance: *8*		*751*		
	>1p	14		0				
	nsp	110		2				
	sp	619		6				
4	serostatus discordance disclosure (correct)	partner disclosed having HIV: *46*		respondent disclosed having HIV: *10*		*56*		*10*
	>1 p	2		2				
	nsp	8		5				
	sp	36		3				
5 + 6	no (or incorrect) serostatus disclosure	*1026*	*27.8%*	*101*	*23.4%*	*1127*	27.3% (23.0%)	
5	serostatus disclosure (incorrect)			*failed serosorting: 20*	*4.6%*			*20*
	>1p			2				
	nsp			5				
	sp			13				
6	no condom, no treatment, no disclosure	*1026*	*27.8%*	*81*	*18.8%*	*1107*	26.8% (22.6%)	*81*
	HIV negative concordance assumption	504	13.6%	30	6.9%			
	>1p	22		3				
	nsp	212		14				
	sp	270		13				
	no reported assumptions	522	14.1%	51	11.8%			
	>1p	43		8				
	nsp	245		24				
	sp	234		19				
Subtotal, missing excluded		*3693*	*100%*	*432*	*100%*	*4125*	100% (84.2%)	*111*
Missing	missing data on disclosure and/or type of partner	612	–	65	–	677	(13.8%)	65
	>1p	14		4				
	nsp	49		9				
	sp	72		13				
	mpd	477		39				
	missing or conflicting information on HIV serostatus					99	(2.0%)	
Total		*4305*		*497*		*4901*	*(100%)*	*176*

nsp = non-steady partner; sp. = steady partner; >1p = more than one partner; mpd = missing partner data

The italic numbers are the total numbers and percentages of participants in the respective hierarchical risk management category

Amongst the 27% (*n* = 1107) of participants who did not report serostatus disclosure, almost half (48%) reported *assuming* seroconcordance with their most recent AI partner. Stratified by partner type, seroconcordance was *assumed* by 53% of respondents reporting CLAI without explicit serostatus disclosure with a steady partner, by 46% of respondents reporting CLAI with a non-steady partner, and by 33% of respondents reporting CLAI with more than one partner (see Table 3).

Two percent of AI events (*n* = 92) were between participants living with HIV and reporting an undetectable viral load due to ART and partners not known to have HIV.

Fourteen percent of all participants (677/4901) had to be excluded from this analysis due to missing information on the most recent AI or missing information on the type of partner(s), and another 2% (*n* = 99) due to an invalid biological sample not allowing for HIV serostatus determination (see Table 3).

In our sample, we were unable to identify a modifying impact of strategic positioning (choosing an insertive or receptive role during CLAI to minimise respective HIV transmission risks) on HIV exposure risks. However, approximately one quarter of these recent AI episodes could not be categorised due to missing information.

#### HIV risk management of participants identified as being HIV antibody positive

Regarding the quantitative distribution of the most recent AI episodes involving study participants identified as HIV antibody positive in the study specimen, a proportion of 13% (*n* = 65) could not be categorised due to missing information (see Table 3). This was due to missing answers regarding condom use, treatment and last viral load, as well as missing or incomplete information on serostatus disclosure with their most recent AI partner.

From the 432 AI episodes that could be categorised, 51% (*n* = 221) were protected primarily by condom use, 21% (n = 92) were rendered less infectious or non-infectious for HIV due to antiretroviral treatment of participants diagnosed with HIV. A considerable proportion of condom-protected episodes (34%; 75/221) involved participants who also reported being treated with ART and having an undetectable viral load. Categories 3, 4 and 5, resembling self-reported HIV serosorting or serostatus disclosure, comprised 38 individuals (9%; see Table 3).

From the 184 study participants with laboratory evidence of HIV infection who did not report condom use or treatment as disclosure-independent HIV risk management during their last AI, we were able to determine HIV serostatus awareness for 119. For 65 (65/184 = 35%) the available data was inconclusive as to their HIV serostatus awareness. Thirty-nine of the 119 (33%) were aware of having HIV, 80 were unaware. From these 39, 8 (21%) reported an HIV-concordant sexual partner at the last AI, and 10 (26%) reported HIV status disclosure to their last AI partner, who was not known to have HIV. According to the data we collected, 21 participants aware of having HIV reported neither condom use, nor a viral load below detection limit, nor HIV serostatus disclosure or HIV serosorting with their last AI partner. Among the 111 evaluable AI episodes (119–8) with potential HIV exposure of the partner and information available on HIV serostatus awareness, disclosure, and type of partner, the majority (80/111) was unaware of having HIV. Most of these had not communicated about their serostatus with their most recent AI partner, yet 20 participants (20/111 = 18%) had unknowingly disclosed an incorrect HIV status. In the 176 episodes of not otherwise protected AI with probable risk of HIV exposures, we could not identify any indications of the use of strategic positioning to minimize transmission risks.

### HIV serostatus disclosure and HIV exposures

**Fig. 1:**
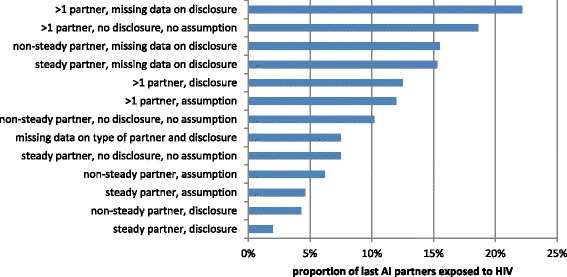
Proportion of potentially HIV-exposed partners during last anal intercourse by type of partner and HIV serostatus disclosure*, Sialon-II bio-behavioural survey, 2013–2014. *excluded: potential exposures associated with disclosed HIV infection status

In the subgroup of the sample that reported HIV serostatus disclosure as HIV risk management (*n* = 827), *unexpected* HIV exposure through CLAI occurred in 20 of 827 (2%) episodes, CLAI in the absence of serostatus disclosure was however associated with HIV exposure in 81 of 1107 (7%) respective episodes. An additional 65 AI episodes of men with undiagnosed HIV infection could not be categorised due to missing data (for a more detailed disaggregation see Fig. 1).

As shown in Fig. 1, the different possible combinations of knowledge of the partner(s) and serostatus disclosure are key factors which determine the probability of being exposed for partners of MSM who have HIV. Essentially, the exposure risk increases with increasing “emotional distance” of the partner(s) and with decreasing communication about HIV serostatus.

There were 96 CLAI episodes involving HIV exposure of *one* partner. These were equally distributed amongst steady and non-steady partners (48 with non-steady partners; see Table 3). In 88 encounters, no (*n* = 70) or no correct (*n* = 18) serostatus disclosure occurred. However, 45 of the 88 episodes were with steady partners where we cannot distinguish between those only holding assumptions about their own and their partners’ HIV status, those having disclosed incorrectly at a previous occasion and those whose serostatus might have changed after having previously been correctly disclosed. In 8 episodes their positive HIV status had been disclosed to the partner according to self-report by HIV positive study participants. For 22 episodes data were missing to classify the communication that had occurred.

Six of the 48 episodes of HIV exposure of a steady partner involved respondents already diagnosed with HIV, all other respondents erroneously believed to be HIV negative (*n* = 23) or had unknown or inconclusive HIV serostatus knowledge (*n* = 19).

Amongst the 48 episodes of HIV exposure of a non-steady partner, 13 respondents were already diagnosed with HIV, 20 respondents erroneously believed not to be infected, and 15 respondents had unknown HIV serostatus knowledge.

## Discussion

More than one third of the study participants who answered all relevant questions and provided a valid specimen for HIV antibody testing reported mutual serostatus disclosure or unilateral disclosure of HIV infection with their most recent AI partner. Most of these disclosures occurred between steady partners, but a moderate proportion also occurred between non-steady partners. Apart from the type of partner, HIV serostatus disclosure was positively associated with the recency of a negative HIV-antibody test result, a positive HIV status, outness regarding sexual orientation, and the lack of condom use. Lack of condom use could also be phrased differently as risk management tactics other than condom use. There was a negative association with perceived anti-gay stigma.

These findings are in line with previous studies analysing factors associated with HIV serostatus disclosure [11, 13–15], and confirm that a higher level of intimacy and trust facilitates serostatus disclosure, as well as the perception that the information on HIV serostatus is relatively recent. The findings also confirm that serostatus disclosure is perceived as an alternative to condom use, predominantly within steady partnerships. The positive association with outness and the negative association with perceived anti-gay stigma point both to the important role of the societal attitudes towards gay men and the political and cultural environment towards homosexuality for the ability of MSM to use preventive services such as HIV testing and to use serostatus knowledge to adopt serostatus-based preventive strategies. Notably, all three cities with significantly worse disclosure levels used RDS for recruitment. RDS was preferentially used in cities where TLS deemed unfeasible due to the lack of gay venues. Therefore confounding between RDS use and other variables with potential impact on HIV serostatus disclosure has to be assumed, and it is possible that RDS sampled a different stratum of MSM populations compared to TLS. MSM recruited in gay venues might be more likely to be “out” or to perceive a gay-friendly environment, compared to those recruited through a network-based method such as RDS, which is more likely to allow the enrolment of a more hidden population of non-gay-identifying MSM [23]. Other factors which may explain the differences between study cities and countries could be internalised homonegativity or structural stigma. We could not explore these factors in our study, but internalised homonegativity has been shown to be negatively associated with being exposed to HIV/STI information for MSM, access to HIV testing, access to STI testing, access to condoms, and little or no experience of gay-related hostility, while structural stigma has been associated with unmet prevention needs, not using testing services, and not discussing one’s sexuality in testing services [24, 25]. It is also conceivable that perceived anti-gay stigma may have an impact on self-esteem and perceived self-efficacy for initiating serostatus communication [26].

Notably, the study participants with undiagnosed HIV infection were significantly less likely to disclose their assumed negative serostatus to their most recent AI partner. The reasons for this finding are unclear and require further research. It could be that these participants already suspect that their assumed serostatus is no longer valid, or it could be that the lack of comfort with serostatus disclosure with their partners and the subsequent assumptions about their partners’ serostatus are risk factors for being exposed and possibly infected by HIV, or it could be that non-disclosure is associated with other risk behaviours such as visiting gay sex venues which may be associated with increased risk for HIV exposure.

The lack of association between serostatus disclosure and venues visited is likely due to a lack of precision of the study’s measurement tool: respondents were asked which type of venues they had visited in the last 3 months (e.g. disco, bar, sauna, sex club etc.) not the venue where they had met their most recent AI partner. Moreover, online dating and chat sites which represent one of the most relevant venues for serostatus disclosure [16, 17] were not queried at all.

There was a lack of association of serostatus disclosure with the frequency of visiting specific gay sex venues such as gay saunas, bars with backrooms, or sex parties in the last 3 months, but we were unable to adequately assess the association between the setting where the most recent AI partner was met and HIV serostatus disclosure with this partner through our questionnaire [17].

Besides condom use, which remains by far the most common risk management tactic amongst our study participants used by 51% of respondents indicating sufficient details about their most recent AI episode, serostatus disclosure, usually followed by condom serosorting was the second most frequently used risk management strategy, mostly used with steady partners. Eighteen percent had CLAI with an apparently seroconcordant partner, and 27% reported CLAI in the absence of other discernible protective behaviours. This differs slightly from the Amsterdam Cohort studies [27] where consistent condom use was reported in 64% of cases, CLAI in 25%, and CLAI with serosorting in 11% of the 2137 follow-up visits. Discrepancies with our findings may be due to shifts in risk management over time (e.g. decreasing condom use or increased use of serosorting), different composition of study samples, and differences in study design and measurement.

A decrease in condom use over time since 2005 amongst MSM in larger US cities has recently been reported, using cross-sectional survey data from the National HIV Behavioral Surveillance (NHBS) [28]. In this survey the authors concluded that decreasing condom use trends were not explained by increasing serosorting or ART.

A group of authors analysing routinely collected behaviour data from HIV testing sites in Seattle over the period 2002–2013 reported that serosorting was increasing concurrent with a decline in non-concordant CLAI, and that consistent condom use remained “fairly constant” [29]. However, the data reported by these authors also supports the view that consistent condom use was declining. Several other studies also suggest an increasing use of serostatus disclosure for HIV risk management amongst MSM [30–32].

In the Amsterdam Cohort studies, MSM who practiced serosorting were less likely to newly acquire HIV infection [adjusted incidence rate ratio (aIRR) = 0.46; 95% CI: 0.13–1.59] than MSM who had CLAI without serosorting. A very similar effect size with a 47% lower risk for serosorting compared to non-concordant CLAI was reported in the Seattle study. Both studies compare well with our findings on HIV exposure rates. In our sample, serostatus disclosure more than halved the HIV exposure risk compared to non-disclosure, yet the exposure risk remained considerable. Whilst differences in seroconversion rates between men practicing serosorting and men practicing CLAI with no discernible protective behaviours in the Amsterdam Cohort were not statistically significant, MSM who consistently used condoms were less likely to seroconvert than MSM who had CLAI (aIRR = 0.37; 95% CI: 0.18–0.77). In the Seattle study, MSM who did not report CLAI had the lowest HIV-test positivity rate when compared to serosorting or non-concordant CLAI.

Analysing the role of serostatus disclosure and concordance for risk reduction amongst the subset of participants with HIV antibodies, establishing seroconcordance or HIV infection disclosure was the primary risk management tactic for 9% (38/432) of the AI episodes, yet an incorrect serostatus due to being unaware of an undiagnosed HIV infection was disclosed in 20 evaluable episodes. Having an undetectable viral load due to ART was the most important reason for reduced HIV exposure in this group. Just 26% (111/432) of the reported AI episodes in the sample of participants with HIV antibody-positive study specimens appeared to be associated with potential HIV exposure of the partner, and in 2% the partner may have been aware of this risk.

### Limitations

Our findings come with several limitations. Whilst we used TLS and RDS as recruitment strategies which represent the current main approaches to conduct bio-behavioural surveys to reduce recruitment bias, the study sample is not (nor was it intended to be) representative for the MSM population of the 13 study cities [33]. Therefore we joined the data and considered it as a convenience sample without applying separate sampling weights for all analyses, nor adjusting for clustering effects. It should be mentioned that RDS was used preferentially in cities where TLS was deemed unfeasible due to the relative lack of gay venues as indicated by the formative research. Therefore confounding between RDS use and other variables with potential impact on HIV serostatus disclosure has to be assumed, in addition to RDS possibly sampling a different stratum of the city population compared to TLS [23]. In addition, neither the laboratory specimen analysis nor the questionnaire were designed to pinpoint the moment of HIV transmission in HIV positive participants. It is therefore possible that the last sexual experience participants reported coincided with the infection.

As in all studies with self-reported behaviours, data may be affected by recall and social desirability bias, particularly since sensitive behaviours like CLAI were explored. The generalisability of our findings is further limited by considerable non-response rates for several of the behaviours analysed. The distribution of risk management tactics for AI episodes reported by HIV antibody-positive respondents could substantially change if the episodes with missing information could be re-categorised. However, since the proportions that could not be categorised were very similar in the subgroups with and without HIV antibodies, there is no indication for a significant HIV status-related non-response bias. We may underestimate serostatus disclosure for people with more than one partner at the most recent AI episode, between steady partners, and with non-steady partners with whom the respondents have sex on a regular basis, because we asked for serostatus disclosure only in the context of the most recent AI and not for all prior serostatus disclosures. Amongst the risk management tactics analysed, we can only assess the failure rate of serostatus disclosure. We are unable to assess failure rates for condom use and for preventive effects of ART on HIV exposure. Since we used closed questions to categorise risk management tactics, it may well be possible that respondents applied other individual measures to reduce or modify HIV transmission risks (e.g. withdrawal before ejaculation). Finally, exposure is not the same as transmission. Additional factors such as viral load, concomitant STIs, frequency of CLAI, and number of AI partners can greatly modify the HIV-exposure associated transmission risk, and these factors are unlikely to be distributed evenly amongst men having CLAI with steady and non-steady partners, and amongst men aware and unaware of having HIV.

## Conclusions

We found that CLAI after mutual serostatus disclosure is associated with a substantially lower risk for HIV exposure than non-disclosure, not using condoms, and not having a partner on effective ART, confirming previous findings [34]. However, incorrect knowledge of the current HIV status could contribute to a large proportion of the current HIV exposures amongst European MSM, both in steady and non-steady partnerships. Within steady partnerships, these exposures will only be preventable either by maintaining consistent condom use or by achieving more accurate serostatus knowledge within steady partnerships through (couple) testing at the onset of the partnership, followed by ‘negotiated safety’ agreements and a testing frequency which is adapted to the frequency of CLAI with concurrent non-steady partners. This will require easily accessible testing services, including HIV self-testing with home tests or home collection testing schemes. It is unlikely that HIV exposures within steady partnerships due to incorrect serostatus disclosure can be addressed by oral HIV chemoprophylaxis (pre-exposure prophylaxis; PrEP) because people tend to feel safe with their steady partners. On the contrary, exposures outside steady partnerships, associated mostly with no HIV serostatus disclosure, could be prevented by oral chemoprophylaxis, as well as by condom use and more frequent and accurate HIV serostatus disclosure. Taking PrEP would also be accompanied by more frequent HIV testing, which would enable early detection of PrEP failures and prompt initiation of treatment.

Based on our findings we would assume that continuing and increasing test promotion for MSM will increase the use of serostatus disclosure for HIV risk management, particularly within steady partnerships and with other partners with whom men have AI repeatedly or on a regular basis. This should be tested and confirmed by future research. Assuming that (consistent) condom use is less prone to unintended HIV exposures than HIV serosorting, future increases or declines of HIV transmission risks amongst MSM will thus partly depend on the strategies that they replace: risks may decline if no discernible risk management has previously been used, risks may increase if current condom users increasingly adopt serosorting instead.

To reach the goal of ending HIV/AIDS as a public health emergency amongst MSM in Europe by 2030, likely a combination of different approaches and strategies will be required: maintaining or improving consistent condom use for AI; early and effective ART after HIV diagnosis; increased HIV testing frequency to improve the accuracy of HIV serostatus awareness; increased mutual HIV serostatus disclosure and serostatus knowledge particularly within steady partnerships; the provision and correct use of oral HIV chemoprophylaxis (PrEP) for men engaging frequently in CLAI with known and untreated HIV-serodiscordant partners or with non-steady partners.

## Additional files

Additional file 1: Study questionnaire. (PDF 235 kb)

Additional file 2: Stata do-files for generation of new secondary variables and data analysis. (TXT 26 kb)

Additional file 3: Sialon-II study dataset, restricted to variables included in the analysis. (CSV 2840 kb)

## Abbreviations

>1p: More than one partner
AI: Anal intercourse
aIRR: Adjusted incidence rate ratio
ART: Anti-retroviral therapy
CI: Confidence interval
CLAI: Condomless anal intercourse
HIV: Human Immunodeficiency Virus
IQR: Interquartile range
MLM: Multilevel logistic regression model
mpd: Missing partner data
MSM: Men who have sex with men
NHBS: National HIV Behavioral Surveillance
nsp: Non-steady partner
OR: Odds ratio
PMM: Predictive mean matching
PrEP: Pre-exposure prophylaxis
RDS: Respondent driven sampling
sp: Steady partner
STI: Sexually transmitted infection
TLS: Time location sampling
WHO: World Health Organization
WHO-ERC: WHO Research Ethics Review Committee
WHO-RP2: WHO Research Project Review Panel
