# Supplementary material for: HIV serostatus knowledge and serostatus disclosure with the most recent anal intercourse partner in a European MSM sample recruited in 13 cities: results from the Sialon-II study
Source: BMC Infect Dis. 2017 Nov 25;17:730. doi: 10.1186/s12879-017-2814-x (PMC5702243; doi:10.1186/s12879-017-2814-x)
Supplement: Supplementary file 1 — Study questionnaire. (PDF 235 kb) [file 12879_2017_2814_MOESM1_ESM.pdf]

This questionnaire is completely anonymous. Please answer all questions.

To answer, put an X inside the box provided ☒.
If you want to change your answer, simply fill in the wrong box completely and cross the correct one.
When asked for a number, put one digit in each space: |_9_|_9_|
In the free text fields, use preferably print.

1. **Which year were you born?** 19 |__|__|

2. Which country were you born in?

[*In the study country*] ☐ 1

Other (specify) ____________________

2a. Which country was your <u>mother</u> born in?

[Same country as study] ☐ 1

Other (specify) ____________________

2b. Which country was your <u>father</u> born in?

[Same country as study] ☐ 1

Other (specify) ____________________

3. Which country do you currently live in?

[*In the study country*] ☐ 1

Other (specify) ____________________

4. Where do you live?

- [*In the study city*] ☐ 1
- Within 100 km of [*the study city*] ☐ 2
- I am a tourist/visitor/commuter from within the same country ☐ 3
- I am a tourist/visitor from another country ☐ 4

5. What is your highest educational qualification?

- No qualification ☐ 1
- CSE ☐ 2
- 'O' level ☐ 3
- 'A' levels ☐ 4
- Higher education (below degree level) ☐ 5
- University degree (e.g. Bachelors, Masters, PhD) ☐ 6
- I prefer not to answer ☐ 9

6a. During the last three months, how many times did you visit the following types of venues? (*Please refer to the list provided to see which venues are included under each type. Please answer each item. If you are not sure, give an estimation.)*

a.	Gay disco/dance club	|__|__|__| times
b.	Gay café	|__|__|__| times
c.	Gay bar	|__|__|__| times
d.	Gay sauna	|__|__|__| times
e.	Gay Sex club	|__|__|__| times
f.	Gay Sex shop	|__|__|__| times

6b. During the last three months, how many times did you visit this particular venue where we are right now. (*If you are not sure, please give an estimation.)*

|__|__|__| times

7. Thinking about all the people who know you (including family, friends and work or study colleagues), what proportion know that you are attracted to men?

- None ☐ 1
- Few ☐ 2
- Less than half ☐ 3
- More than half ☐ 4
- All or almost all ☐ 5
- I prefer not to answer ☐ 9

8. In your experience, what is most people's attitude towards gays or bisexuals in the following contexts? *(please answer each item)*

		Very negative	Negative	Neither negative nor positive	Positive	Very positive
a.	Work/School	☐ 1	☐ 2	☐ 3	☐ 4	☐ 5
b.	Parents	☐ 1	☐ 2	☐ 3	☐ 4	☐ 5
c.	Friends/Acquaintances	☐ 1	☐ 2	☐ 3	☐ 4	☐ 5

9. Do you know where you can go if you wish to receive an HIV test?

Yes ☐ 1

No ☐ 2

I prefer not to answer ☐ 9

From this point onwards we would like you to focus on the past 12 months.

10. In the last 12 months, have you been given condoms - e.g. through an outreach service, drop-in center or sexual health clinic (excluding this SIALON II project)?

Yes ☐ 1
No ☐ 2 ➡ *Go to question 12*
I prefer not to answer ☐ 9 ➡ *Go to question 12*

11. If you have been given condoms in the last 12 months, where did you get them from (excluding this SIALON II project)?

		Yes	No	I prefer not to answer
a.	At drop-in centre, sexual-health clinic, health care facility	☐ 1	☐ 2	☐ 9
b.	By an outreach service/ gay/HIV/other association	☐ 1	☐ 2	☐ 9
c.	At saunas, clubs, discos, bars	☐ 1	☐ 2	☐ 9
d.	At other type venues or settings	☐ 1	☐ 2	☐ 9

12. In the last 12 months, have you been tested for sexually transmitted infections other than HIV?

Yes ☐ 1
No ☐ 2 ➡ *Go to question 14*
I prefer not to answer ☐ 9 ➡ *Go to question 14*

13. In the last 12 months, have you been diagnosed with the following *(please answer each item)*:

		Yes	No	I do not know	I prefer not to answer
a.	Syphilis	☐ 1	☐ 2	☐ 8	☐ 9
b.	Gonorrhoea	☐ 1	☐ 2	☐ 8	☐ 9
c.	Chlamydia	☐ 1	☐ 2	☐ 8	☐ 9
d.	Anogenital Warts	☐ 1	☐ 2	☐ 8	☐ 9
e.	Genital herpes	☐ 1	☐ 2	☐ 8	☐ 9
f.	Lymphogranuloma venereum (LGV)	☐ 1	☐ 2	☐ 8	☐ 9
g.	Urethral outflow/itching	☐ 1	☐ 2	☐ 8	☐ 9
h.	Hepatitis B	☐ 1	☐ 2	☐ 8	☐ 9
i.	Hepatitis C	☐ 1	☐ 2	☐ 8	☐ 9

14. Have you been tested for HIV in the last 12 months?

Yes ☐ 1

No ☐ 2 ➡ *Go to question 16*

I prefer not to answer ☐ 9 ➡ *Go to question 16*

15. If you had an HIV test in the last 12 months, did you receive the result of that test?

Yes ☐ 1

No ☐ 2

I prefer not to answer ☐ 9

From this point onwards we would like you to focus on the <u>last 6 months</u> and your <u>male non-steady partners</u>.

"Non-steady partners"** refer to men you have had sex with once only, and men you have sex with more than once but who you don't think of as a steady partner (including one night stands, anonymous and casual partners, sex buddies)**.

*By **"sex"** we mean any kind of sex involving physical contact with another person, including oral sex and mutual masturbation.*

16. In the last 6 months, how many male *non-steady* partners have you had sex with? *(If you are not sure, please give an estimation.)*

Number of male non-steady partners (0 if none) |__|__|__|

I prefer not to answer ☐ 999

17. In the last 6 months, how many male *non-steady* partners have you had <u>anal intercourse</u> with? *(If you are not sure, please give an estimation.)*

Number of male non-steady partners (0 if none) |__|__|__|

I prefer not to answer ☐ 999

18. In the last 6 months, how many male *non-steady* partners have you had <u>unprotected (without condom) anal intercourse</u> with? *(If you are not sure, please give an estimation.)*

Number of male non-steady partners (0 if none) |__|__|__|

I prefer not to answer ☐ 999

19. How many of those you had <u>unprotected (without condom) anal intercourse</u> with were:

HIV positive (0 if none) |__|__|__|

With unknown HIV status (0 if none) |__|__|__|

HIV negative (0 if none) |__|__|__|

I prefer not to answer ☐ 999

Sialon II

From this point onwards we would like you to focus on the <u>last 6 months</u>
and your <u>male steady partners</u>.
*"**Steady partners**" refer to boyfriends or husbands that mean you are not "single", but not to partners who are simply sex buddies.*
*By **"sex"** we mean any kind of sex involving physical contact with another person, including oral sex and mutual masturbation.*

20. In the last 6 months, how many male *steady* partners have you had sex with? *(If you are not sure, please give an estimation)*

Number of male steady partners (0 if none) |__|__|__|

I prefer not to answer ☐ 999

21. In the last 6 months, how many male *steady* partners have you had <u>anal intercourse</u> with? *(If you are not sure, please give an estimation)*

Number of male steady partners (0 if none) |__|__|__|

I prefer not to answer ☐ 999

22. In the last 6 months, how many male *steady* partners have you had <u>unprotected (without condom) anal intercourse</u> with? *(If you are not sure, please give an estimation)*

Number of male steady partners (0 if none) |__|__|__|

I prefer not to answer ☐ 999

23. In the last 6 months, how many of those you had <u>unprotected (without condom) anal intercourse</u> with were? *(If you are not sure, please give an estimation)*

HIV positive (0 if none) |__|__|__|

With unknown HIV status (0 if none) |__|__|__|

HIV negative (0 if none) |__|__|__|

I prefer not to answer ☐ 999

24. <u>In the last 6 months</u>, how many <u>female</u> partners (non-steady and steady) have you had sex with? *(any kind of sex; if you are not sure, please give an estimation.)*

Number of female partners (0 if none) |__|__|__| ➡ *If NONE, go to question 26*

I prefer not to answer ☐ 999

25. <u>In the last 6 months</u>, did you have any unprotected (without condom) anal or vaginal intercourse with a <u>woman</u>?

Yes ☐ 1

No ☐ 2

I prefer not to answer ☐ 9

From this point onwards, we would like you to focus on the last time you had anal intercourse with a male partner.

26. Last time you had anal intercourse with a male partner, who did you have it with?

- One steady partner ☐ 1
- One non-steady partner ☐ 2
- More partners at once (threesome, group sex, etc.) ☐ 3
- I have never had anal intercourse ☐ 4 ➡ *Go to question 31*
- I prefer not to answer ☐ 9

27. Last time you had anal intercourse, were you …

		Yes	No	I prefer not to answer
a.	penetrated (fucked) **with** a condom?	☐ 1	☐ 2	☐ 9
b.	penetrating (fucking) **with** a condom?	☐ 1	☐ 2	☐ 9
c.	penetrated (fucked) **without** a condom?	☐ 1	☐ 2	☐ 9
d.	penetrating (fucking) **without** a condom?	☐ 1	☐ 2	☐ 9

28. Last time you had anal intercourse, what did you think about your partner/s HIV status before having sex? *(In case of multiple partners, tick as many as apply.)*

- I thought he was HIV negative ☐ 1
- I knew he was HIV negative ☐ 2
- I thought he was HIV positive ☐ 3
- I knew he was HIV positive ☐ 4
- I knew he was unsure about his HIV status ☐ 5
- I didn't have any thoughts about his HIV status ☐ 6
- I do not remember ☐ 8
- I prefer not to answer ☐ 9

29. Last time you had anal intercourse, what did you tell your partners/s about your HIV status before having sex? *(In case of multiple partners, tick as many as apply.)*

- I told him I didn't know my HIV status ☐ 1
- I told him I was HIV negative ☐ 2
- I told him I was HIV positive ☐ 3
- I said nothing about my HIV status ☐ 4
- I told him I was unsure about my HIV status ☐ 5
- I do not remember ☐ 8
- I prefer not to answer ☐ 9

30. Before or during your last anal sexual intercourse, did you use the following? *(Please answer each item.)*

		Yes	No	I prefer not to answer
a.	Alcohol	☐ 1	☐ 2	☐ 9
b.	Poppers	☐ 1	☐ 2	☐ 9
c.	Ecstasy	☐ 1	☐ 2	☐ 9
d.	Viagra/Camagra/Ciallis	☐ 1	☐ 2	☐ 9
e.	Hashish/marijuana	☐ 1	☐ 2	☐ 9
f.	Cocaine	☐ 1	☐ 2	☐ 9
g.	Amphetamine	☐ 1	☐ 2	☐ 9
h.	GHB	☐ 1	☐ 2	☐ 9
i.	Other (specify: ______________________)	☐ 1	☐ 2	☐ 9

31. In which year did you have your last HIV test in order to detect your HIV-status? *(Please give an approximate year if you do not remember the exact year.)*

Year |__|__|__|__|

I have never been tested ☐ 8 ➡ *Go to question 35*

I prefer not to answer ☐ 9 ➡ *Go to question 35*

32. What was the result of your last HIV test?

HIV positive (I have HIV) ☐ 1

HIV negative (I do not have HIV) ☐ 2

Indeterminate ☐ 3

I did not get the result of the test ☐ 4

I prefer not to answer ☐ 9

} ➡ *Go to question 35*

33. In case you are living with HIV: are you currently taking drugs for treatment for HIV (known as antiretroviral, ART, HAART)?

Yes ☐ 1

No ☐ 2

I prefer not to answer ☐ 9

34. In case you are living with HIV: what was your last viral load?

Undetectable ☐ 1

Detectable ☐ 2

I do not remember / I don't know / I didn't get the result of the test ☐ 3

I prefer not to answer ☐ 9

35. Have you ever injected drugs? (*Drugs injected for medical purposes or treatment of an illness do not count.)*

Yes ☐ 1
No ☐ 2
I prefer not to answer ☐ 9

36. How satisfied are you with your sex life?

Very satisfied ☐ 1
Somewhat satisfied ☐ 2
Somewhat unsatisfied ☐ 3
Very unsatisfied ☐ 4
I prefer not to answer ☐ 9

THANK YOU FOR YOUR KIND COLLABORATION!

PLEASE DO NOT COMPLETE THIS SECTION. FOR DATA COLLECTORS ONLY

Date (dd-mm-yy) |__|__| - |__|__| - |__|__|__|__|
Time (hh:mm) __ __:__ __

Location ____________________________

Interviewer's code |__|__|__|

bar code tag here
